# Risk Factors for Gout in Taiwan Biobank: A Machine Learning Approach

**DOI:** 10.2147/JIR.S490821

**Published:** 2024-11-26

**Authors:** Yu-Ruey Liu, Oswald Ndi Nfor, Ji-Han Zhong, Chun-Yuan Lin, Yung-Po Liaw

**Affiliations:** 1 College of Information and Electrical Engineering, Asia University, Taichung, 413, Taiwan; 2 Department of Emergency Medicine, Cheng Ching General Hospital, Taichung, Taiwan; 3 Department of Computer Science and Information Engineering, Asia University, Taichung, 413, Taiwan; 4 Department of Public Health and Institute of Public Health, Chung Shan Medical University, Taichung, Taiwan; 5 Institute of Medicine, Chung Shan Medical University, Taichung, Taiwan; 6 Department of Medical Imaging, Chung Shan Medical University Hospital, Taichung, Taiwan

**Keywords:** risk prediction, gout, machine learning, artificial intelligence

## Abstract

**Purpose:**

We assessed the risk of gout in the Taiwan Biobank population by applying various machine learning algorithms. The study aimed to identify crucial risk factors and evaluate the performance of different models in gout prediction.

**Patients and Methods:**

This study analyzed data from 88,210 individuals in the Taiwan Biobank, identifying 19,338 cases of gout and 68,872 controls. After data cleaning and propensity score matching for gender and age, the final analytical sample comprised 38,676 individuals (19,338 gout cases and 19,338 controls). Five machine learning models were used: Bayesian Network (BN), Random Forest (RF), Gradient Boosting (GB), Logistic Regression (LR), and Neural Network (NN). The predictive performance was evaluated using a split dataset (80% training set and 20% test set).

**Results:**

Variable importance analysis was performed to identify key variables, with uric acid and gender emerging as the most influential risk factors. Descriptive data highlighted significant differences between the control group and gout patients, with a higher prevalence of gout in men (51.36% vs 48.64%). Both the RF and GB demonstrated high performance across multiple metrics, with RF consistently achieving a high area under the curve (AUC) of 0.986 to 0.987, alongside excellent sensitivity (0.945–0.947) and specificity (0.998–0.999). GB also performed robustly, with AUC values around 0.987–0.988 and maintaining high sensitivity (0.944–0.950) and specificity (0.995–0.999) across different model variations. The F1 scores for both models (GB and RF) indicate strong predictive capabilities, with values around 0.971–0.972.

**Conclusion:**

The RF and GB demonstrated exceptional accuracy in predicting gout status, particularly when incorporating genetic data alongside clinical factors. These findings underscore the potential for integrating machine learning models with genetic information to enhance gout prediction accuracy in clinical practice.

## Introduction

Gout is a complex inflammatory condition primarily caused by hyperuricemia leading to monosodium urate (MSU) crystal deposition in joints and other tissues. Its clinical presentation includes acute painful flares and chronic complications.^1^ The disease etiology is multifactorial, involving genetic predispositions, lifestyle factors such as diet and alcohol consumption, and certain medical conditions that affect uric acid metabolism.^2^,^3^ Gout is associated with various comorbidities, including cardiovascular diseases and renal impairment, which can complicate its management and exacerbate patient morbidity.^1^,^4^

Gout is a prevalent and debilitating rheumatic disease with an increasing global incidence, especially in Pacific and developed countries.^5^ The Taiwanese population, like many others, faces the growing burden of gout, with a reported prevalence of approximately 6.24%.^6^ The high recurrence rate linked to this condition leads to diminished health-related quality of life^7^,^8^ and heightened financial strain, particularly for individuals unable to manage it effectively.^9^

On a global scale, there were 10,016,336 reported cases in 2023, which is anticipated to rise to approximately 12,082,807 by 2035.^7^ Understanding the risk factors and developing effective prediction models for gout is essential for proactive management and prevention. In this context, the use of data-driven methods, particularly machine learning (ML), has gained prominence as a powerful tool for disease risk assessment.^10^

The conventional approach (that is, traditional statistical techniques, including logistic regression models) to assessing gout risk often relies on well-established clinical risk factors such as age, gender, genetics, lifestyle, and serum urate levels.^11^ While these factors provide valuable insights, they may not fully capture the complexity of the disease, and there is an increasing need for more accurate, individualized risk assessments. According to previous research findings,^10^,^12^ conventional models, which greatly rely on multivariate regression models overly simplify the links between disease risk factors and outcomes excessively, reducing the prediction accuracy. This is where machine learning techniques come into play.

Machine learning, with its capacity to analyze vast datasets, uncover intricate nonlinear relationships between variables, and generate predictive models, offers a promising alternative to conventional methods. Through the application of ML algorithms, researchers and clinicians can explore a broader spectrum of risk factors, potentially identifying subtle associations and interactions that might be missed by traditional statistical approaches. The goal of this scholarly article is to provide a comprehensive comparative analysis of machine learning and conventional approaches for assessing gout risk among the Taiwanese population. By evaluating the strengths and limitations of each approach, we intend to shed light on their respective roles in enhancing the precision and efficacy of gout risk assessment.

The emergence of machine learning in disease risk assessment marks a pivotal moment in the evolution of medical diagnostics and preventive medicine. Nevertheless, we recognize the importance of weighing its potential benefits against the established strengths of conventional approaches, which have long been the cornerstone of clinical practice. Machine learning classifiers have been successfully used to predict diseases risk including but not limited to heart diseases,^13^ diabetes^14–16^ and its complications,^17^ and others.

An earlier study^18^ explored gout staging through machine learning methodologies and suggested further research to enhance the precision of diagnostic models. In light of this, we explored machine learning approaches to determine their performance in gout risk assessment.

## Materials and Methods

### Data Source and Disease Identification

The study data were collected from the TWB dataset, which had phenotypic and genetic data collected from Han Chinese individuals between the ages of 30 and 70. These individuals had completed a standardized questionnaire on physical, sociodemographic, and past medical history. The database is a large-scale population-based biobank managed by the National Health Research Institutes (NHRI) of Taiwan. Established in 2010, the biobank aims to collect comprehensive health and genetic data from the Taiwanese population to support research on various diseases and public health issues. It includes biological samples, health records, and lifestyle information from over 200,000 participants. Its primary purpose is to facilitate research into the genetic, environmental, and lifestyle factors contributing to health and disease in Taiwan. By providing a rich resource for researchers, the Taiwan Biobank plays a crucial role in advancing precision medicine and improving public health strategies in the region.

Genotyping in the biobank was conducted using the C2-58 Axiom Genome-Wide TWB 2.0 Array, developed specifically for people of Taiwanese descent. The dataset included information on gout diagnosis and related variables. We extracted data for 88,347 individuals assessed at the biobank centers from 2008 to 2019.

In Taiwan, the diagnosis of gout typically involves a combination of clinical examination and specific diagnostic criteria. While clinical examination of monoarticular arthritis is indeed a significant aspect of the diagnosis, it is not the sole method. The diagnosis is often supported by the presence of MSU crystals in synovial fluid or tophi, which provides definitive evidence of gout.^19^ In our research, gout was primarily identified through self-reporting, as indicated by the question, “Have you or your family members (biological parents or blood-related siblings) ever been diagnosed with gout?” A response of “yes” signified the presence of the condition, while a response of “no” indicated its absence. Additionally, disease diagnosis was supported by the measurement of uric acid levels; specifically, a uric acid level of 7 mg/dL or higher in men and 6 mg/dL or higher in women. An individual was considered to have gout if either the self-report response was “yes” or if the uric acid levels were elevated. Comprehensive descriptions of the lifestyle factors and other variables considered in our study have been documented in previous reports.^20^,^21^

### Data Processing and Model Development

**Figure 1:**
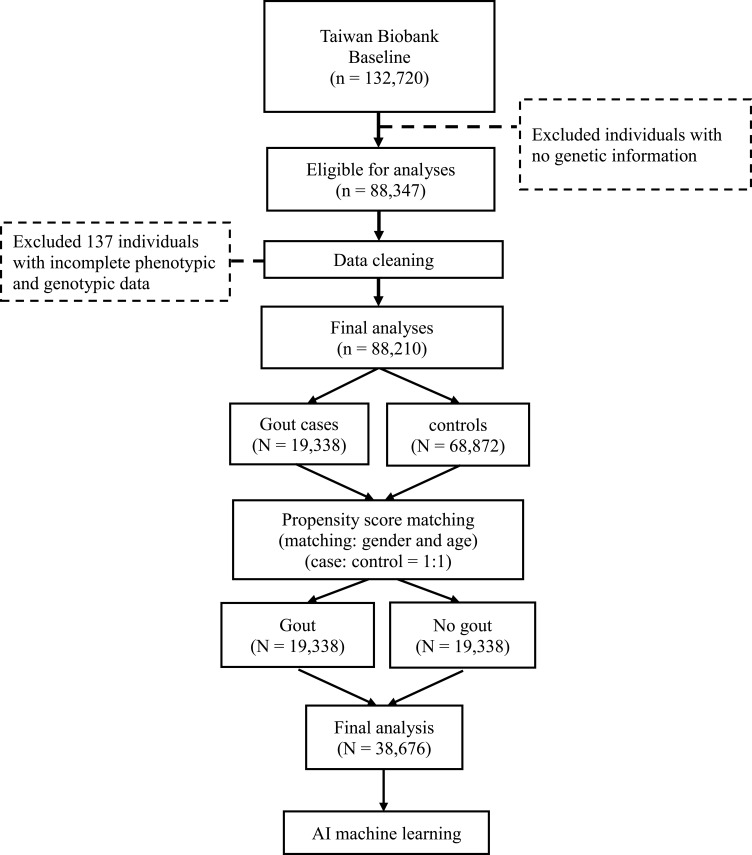
Data processing pipeline.

Our data were cleaned and subjects with and without gout (n = 88,210) were extracted. Figure 1 illustrates the data processing pipeline. From the above subjects, those with missing values (n = 137) were excluded. The algorithms used to assess matrices like the Youden index, AUC, sensitivity, and specificity included BN, RF, GB, LR, and NN. Details of these algorithms have been described elsewhere.^22^ These algorithms were selected based on their established efficacy in previous research.^23^ The traditional model we employed is logistic regression, a widely recognized statistical technique for binary classification tasks, which serves as a benchmark against which we compared the performance of the machine learning algorithms. In terms of performance metrics, we focused on the following key indicators: accuracy (the overall proportion of correct predictions (both true positives and true negatives) among all observations), sensitivity or recall (which indicates the model’s ability to correctly identify positive cases (true positives) out of all actual positive cases), specificity (which measures the ability of the model to correctly identify negative cases (true negatives) out of all actual negative cases), and F1 score (the harmonic mean of precision and recall, providing a balance between the two).

### Statistical Analysis

We utilized SAS Software version 9.4 (SAS Institute, Cary, NC, USA) and PLINK 1.90 beta software^24^ for data management. Continuous and discrete variable distributions were assessed using *t*-tests and chi-square tests. Categorical variables were presented as count and percentages %, while continuous variables as means and standard deviations (SD). We estimated propensity scores using the PROC LOGISTIC and PROC SQL was used to cases and controls in a 1:1 ratio, ensuring precise alignment on gender and age to minimize bias. We checked balance between the case and control group by calculating the Standardized Mean Differences (SMD). We employed logistic regression to estimate the AUC for both internal and external factors in relation to gout.

For the development of AI models, we harnessed SAS^®^ Viya^®^ version 3.5 (SAS Institute Inc., Cary, NC, USA). In this study, we considered various supervised learning models described above. We divided the dataset into training (80%) and test (20%) partitions before constructing machine learning models. The AUC, which signifies the area under the Receiver Operating Characteristic (ROC) curve, was used to assess model performance. An AUC value close to 1 indicates a near-perfect model. To select the best model in machine learning model comparison, we calculated Youden’s Index (also known as Youden’s J statistic).

In both basic statistics and machine learning models, we designated gout as the outcome and target variable. Adjusted covariates included age, gender, body mass index, smoking status, alcohol consumption, exercise habits, uric acid, creatinine, high-density lipoprotein (HDL)-cholesterol, low-density lipoprotein (LDL)-cholesterol, triglycerides (TG), and ABCG2 rs2231142. Different covariates were considered in various models. Moreover, we incorporated a variety of genetic models to enhance the analysis and interpretation of gout risk, potentially yielding more meaningful insights into the role of genetics in health outcomes.

## Results

**Figure 2:**
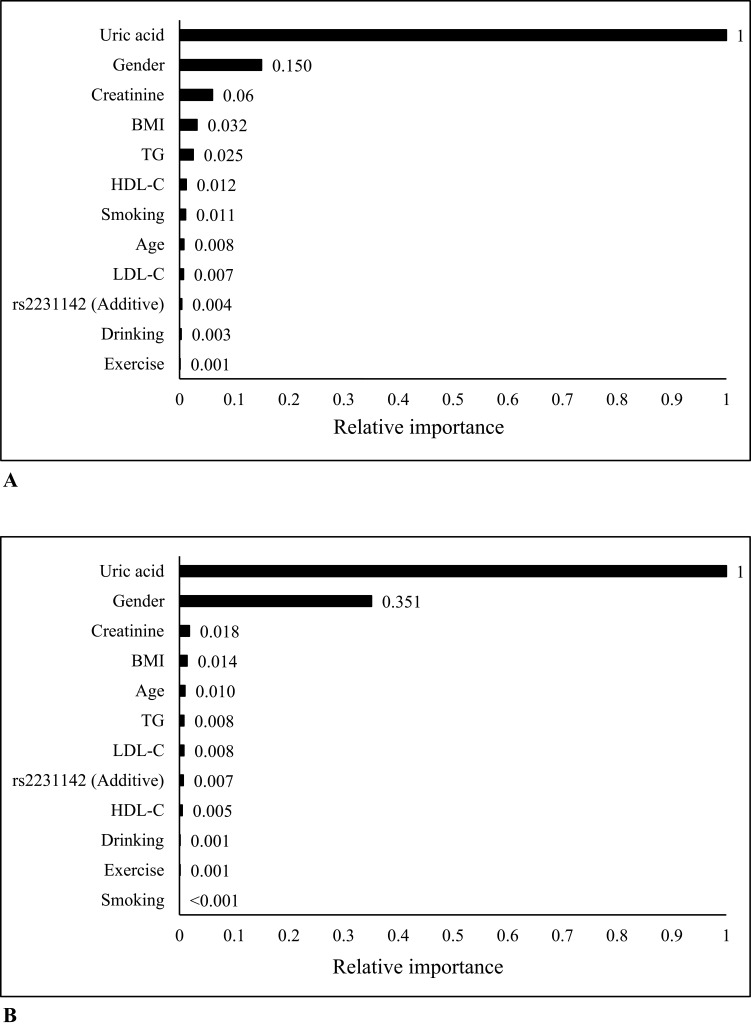
(**A** and **B**) show the variables’ importance in the additive model before and after propensity score matching. The champion model before propensity score matching was Random Forest and Gradient Boosting after matching.

As shown in Figure 1, this study analyzed data from 88,210 individuals in the TWB, identifying 19,338 cases of gout and 68,872 controls. After data cleaning and propensity score matching for gender and age, the final analytical sample comprised 38,676 individuals (19,338 gout cases and 19,338 controls). At baseline and prior to propensity score matching, the mean age (SD) was 49.85 (10.78) years for the controls and 51.56 (11.01) years for the cases with gout (p<0.001) (Table 1). There were more men with gout compared to women (51.36% vs 48.64%). Before matching, there were significant differences in all variables between cases and controls (p<0.001). The variable importance (determined using the tree-based methods including the Mean Decrease Impurity [Gini Importance]) for models with and without genetic marker is shown in Figure 2A and B. There were 12 most important variables, with uric acid and gender emerging as the most important risk factors for gout.

**Table 1:** Basic Characteristics of Case and Control Populations Before and After Propensity Score Matching

Before Propensity Score Matching				After Propensity Score Matching		
	No gout (n = 68,872)	Gout (n = 19,338)	P-value	No gout (n = 19,338)	Gout (n = 19,338)	P-value
**Gender**			<0.001			1.000
Female	50,652 (73.55%)	9406 (48.64%)		9406 (48.64%)	9406 (48.64%)	
Male	18,220 (26.45%)	9932 (51.36%)		9932 (51.36%)	9932 (51.36%)	
**Age (y)**	49.85±10.78	51.56±11.01	<0.001	51.56±11.01	51.56±11.01	1.000
**Body mass index** (kg/m^2^)	23.56±3.51	26.43±4.00	<0.001	24.06±3.53	26.43±4.00	<0.001
**Smoking (n, %)**			<0.001			<0.001
No	57,942 (84.13%)	14,041 (72.61%)		14,536 (75.17%)	14,041 (72.61%)	
Yes	10,930 (15.87%)	5297 (27.39%)		4802 (24.83%)	5297 (27.39%)	
**Drinking (n, %)**			<0.001			<0.001
No	64,418 (93.53%)	16,703 (86.37%)		17,360 (89.77%)	16,703 (86.37%)	
Yes	4454 (6.47%)	2635 (13.63%)		1978 (10.23%)	2635 (13.63%)	
**Exercise (n, %)**			<0.001			0.837
No	41,461 (60.20%)	11,028 (57.03%)		11,048 (57.13%)	11,028 (57.03%)	
Yes	27,411 (39.80%)	8310 (42.97%)		8290 (42.87%)	8310 (42.97%)	
**Uric acid (mg/dL)**	4.83±0.95	7.23±1.12	<0.001	5.14±0.99	7.23±1.12	<0.001
**Creatinine (mg/dL)**	0.67±0.25	0.84±0.39	<0.001	0.74±0.28	0.84±0.39	<0.001
**Cholesterol (mg/dL)**						
High density lipoprotein	56.66±13.49	48.92±11.78	<0.001	54.39±13.49	48.92±11.78	<0.001
Low density lipoprotein	119.40±31.20	126.70±33.07	<0.001	121.00±31.58	126.70±33.07	<0.001
Triglycerides (mg/dL)	104.10±81.08	156.50±129.10	<0.001	114.80±95.66	156.50±129.10	<0.001
**ABCG2 rs2231142**						
**Additive**			<0.001			<0.001
GG	34,267 (49.75%)	7270 (37.59%)		9858 (50.98%)	7270 (37.59%)	
GT	28,628 (41.57%)	9297 (48.08%)		7903 (40.87%)	9297 (48.08%)	
TT	5977 (8.68%)	2771 (14.33%)		1577 (8.15%)	2771 (14.33%)	
**Dominant**			<0.001			<0.001
GG	34,267 (49.75%)	7270 (37.59%)		9858 (50.98%)	7270 (37.59%)	
GT+TT	34,605 (50.25%)	12,068 (62.41%)		9480 (49.02%)	12,068 (62.41%)	
**Recessive**			<0.001			<0.001
GG+GT	62,895 (91.32%)	16,567 (85.67%)		17,761 (91.85%)	16,567 (85.67%)	
TT	5977 (8.68%)	2771 (14.33%)		1577 (8.15%)	2771 (14.33%)	

**Notes**: GG, GT, and TT are the ABCG2 rs2231142 genotypes. Categorical variables: n (%). Continuous variables: Mean ± standard deviation.

Table 2 shows the performance metrics for the test data after propensity score matching.

**Table 2:** Performance Metrics for the Test Data After Propensity Score Matching

Algorithm	KS (Youden)	AUC	Sensitivity	Specificity	Accuracy	F1
Model 1^a^						
Bayesian network	0.634	0.908	0.777	0.858	0.817	0.809
Random forest	0.945	0.986	0.947	0.998	0.972	0.972
Gradient boosting	0.944	0.987	0.944	0.999	0.972	0.971
Logistic regression	0.916	0.975	0.935	0.981	0.958	0.957
Neural network	0.926	0.978	0.942	0.984	0.963	0.962
Model 1^b^						
Bayesian network	0.645	0.911	0.810	0.835	0.822	0.820
Random forest	0.945	0.987	0.947	0.998	0.972	0.972
Gradient boosting	0.945	0.987	0.950	0.995	0.973	0.972
Logistic regression	0.910	0.976	0.928	0.982	0.955	0.954
Neural network	0.927	0.979	0.942	0.984	0.963	0.962
Model 1^c^						
Bayesian network	0.647	0.911	0.815	0.832	0.824	0.822
Random forest	0.944	0.987	0.945	0.999	0.972	0.971
Gradient boosting	0.944	0.988	0.945	0.999	0.972	0.971
Logistic regression	0.913	0.976	0.930	0.983	0.957	0.955
Neural network	0.926	0.978	0.943	0.983	0.963	0.962
Model 1^d^						
Bayesian network	0.640	0.909	0.770	0.870	0.820	0.810
Random forest	0.945	0.987	0.946	0.999	0.972	0.972
Gradient boosting	0.945	0.987	0.947	0.998	0.973	0.972
Logistic regression	0.910	0.976	0.928	0.981	0.955	0.954
Neural network	0.927	0.979	0.942	0.985	0.963	0.962

**Notes**: ^a^Model 1: adjusted for gender, age, BMI, smoking, drinking, exercise, uric acid, creatinine, HDL, LDL, and TG. ^b^Model 2: adjusted for gender, age, BMI, smoking, drinking, exercise, uric acid, creatinine, HDL, LDL, TG, and rs2231142 (additive). ^c^Model 3: adjusted for gender, age, BMI, smoking, drinking, exercise, uric acid, creatinine, HDL, LDL, TG, and rs2231142 (dominant). ^d^Model 4: adjusted for gender, age, BMI, smoking, drinking, exercise, uric acid, creatinine, HDL, LDL, TG, and rs2231142 (recessive).

**Abbreviation**: KS, Kernel Smoothing approach.

**Figure 3:**
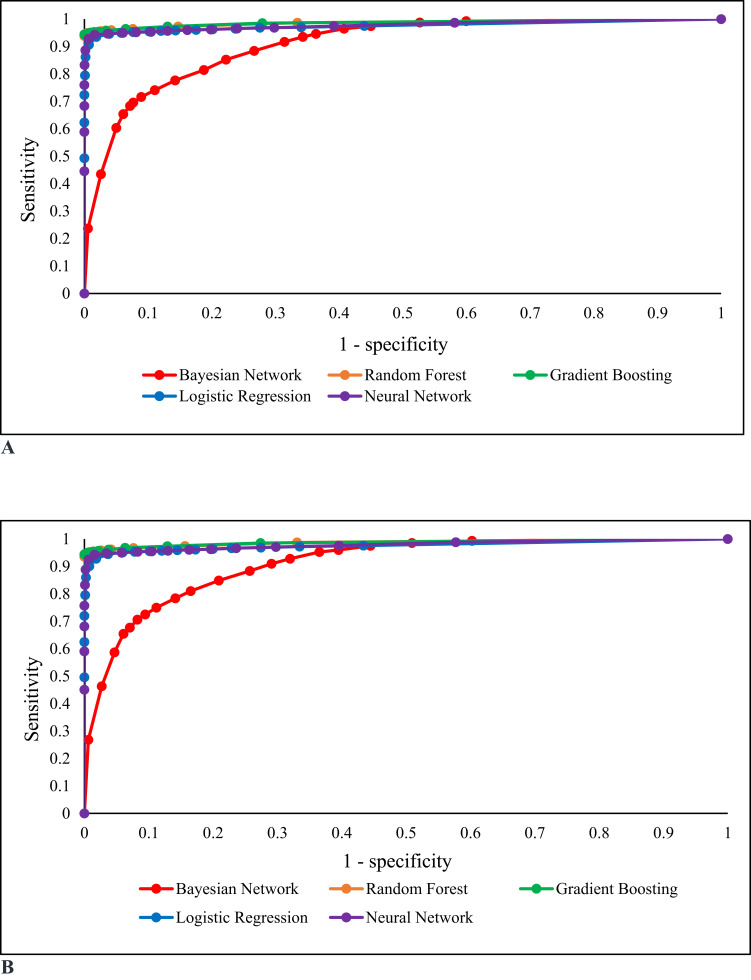
(**A** and **B**) show ROC curves for model 1 (which adjusted for 11 core baseline variables including gender, age, BMI, smoking, drinking, exercise, uric acid, creatinine, HDL, LDL, and TG) and Model 2 (which adjusted for both baseline variables alongside a well-established risk locus (*ABCG2* rs2231142) associated with gout). The champion model was Random Forest for 3A and Gradient Boosting for 3B.

Overall, our dataset was split into a 20% test set and an 80% training set. Across various models displayed in the table, the Random Forest and Gradient Boosting demonstrated superior accuracy (Figure 3A). The RF achieved an AUC of 0.986, sensitivity of 0.947, specificity of 0.998, and F1 score of 0.972. Similarly, GB performed comparably with an AUC of 0.987, sensitivity of 0.944, specificity of 0.999, and F1 score of 0.971, highlighting its robust predictive capability even without genetic information.

Incorporating the ABCG2 rs2231142 risk locus improved prediction metrics marginally across both models (Figure 3B). For instance, in Model 1b (additive adjustment), RF achieved an AUC of 0.987, with a sensitivity of 0.947, specificity of 0.998, and an F1 score of 0.972. GB, in this configuration, also reached an AUC of 0.987 with a sensitivity of 0.950, specificity of 0.995, and F1 score of 0.972.

Across all adjustments (Models 1a to 1d), both RF and GB maintained AUCs above 0.986 and high sensitivity and specificity scores, confirming these models as highly effective predictors of gout. The F1 scores for both models consistently exceeded 0.97, indicating balanced precision and recall.

## Discussion

Our study applied machine learning models to predict gout using clinical and genetic data from a large Taiwanese cohort, with notable findings supporting the efficacy of machine learning in identifying individuals at risk of gout. Key insights from our analyses reveal that RF and GB were the top-performing models, achieving high predictive metrics, even when accounting solely for baseline clinical variables. Moreover, we observed a notable gender disparity among gout patients, with a higher prevalence in men (51.56%) than women (49.85%). Earlier research^7^ demonstrated a rising trend in disease prevalence from 1990 to 2019, and their predictive model based on the age-period-cohort (APC) suggested that this upward trajectory is likely to persist.

Our results indicate that gout risk can be robustly predicted using clinical data alone, with the RF model achieving an AUC of 0.986 and GB reaching an AUC of 0.987. These metrics underscore the models’ capability to differentiate gout cases from non-cases with high sensitivity and specificity, supporting their potential use as diagnostic or screening tools in clinical settings. Notably, integrating genetic information (ie, the ABCG2 rs2231142 risk allele) provided a marginal improvement across all model performance indicators. The consistency of high F1 scores (≥0.97) across models highlights a balanced capacity for both sensitivity and precision. This balance is particularly valuable in a clinical environment, where overdiagnosis and missed cases both pose risks. The ability to maintain such predictive power even after adjustments for gender and age through propensity score matching further strengthens the reliability of these models across diverse demographic groups.

In a previous investigation,^25^ the accuracy of the machine learning method for identifying gout flares was validated, demonstrating enhanced sensitivity and specificity compared to earlier studies. Our study builds upon the growing body of literature utilizing machine learning for disease prediction, specifically in the context of gout. However, to our knowledge, no predictive model has been established for gout, especially in Taiwan. Several studies investigating machine learning models have concentrated on hyperuricemia, with separate observations. For instance, one of them^26^ found that the decision tree performed better than other models. Notably, XG Boost demonstrated superior performance in some of the studies.^27^,^28^ Conversely, another study reported similar predictive efficacy among the decision tree, random forest, and logistic regression approaches.^29^

The utilization of machine learning technologies offers the potential to analyze vast datasets, identify intricate patterns, and to tailor interventions, thereby advancing our ability to proactively address the evolving landscape of gout and optimize patient outcomes. In this research, we found that the Random Forest and Gradient Boosting algorithms demonstrated exceptional accuracy in predicting gout status. The robust performance of these algorithms across different models underscores their versatility and reliability in gout risk prediction. Nevertheless, a recent investigation comparing predictive models for tophi in individuals with gout revealed that the logistic regression model outperformed various machine learning models.^30^ This conclusion was drawn after a comprehensive analysis involving the assessment of AUC, decision curve analysis (DCA), calibration curves, and precision-recall (PR) curves.

The identification of influential factors, including sex, age, BMI, lifestyle factors (smoking, drinking, exercise), and biochemical markers (uric acid, creatinine, HDL-cholesterol, LDL-cholesterol, TG), substantiates the multifactorial nature of gout development. Notably, the integration of the ABCG2 rs2231142 risk locus, a well-established genetic factor in gout pathogenesis, aligns with studies showing its role in elevating serum uric acid levels, thereby exacerbating gout risk. However, while previous studies have focused on traditional statistical methods, our study highlights the added benefit of machine learning models in handling complex, high-dimensional data with higher predictive accuracy.

The predictive accuracy of especially the RF and GB models suggests practical applications in clinical decision-making. These models could serve as non-invasive, early screening tools, especially in populations with high baseline risks (for instance, individuals with elevated BMI or uric acid levels). Furthermore, given the incremental benefit of including genetic data, targeted genetic testing may be beneficial in specific high-risk populations to further refine predictions. Implementing these models in clinical settings could help identify at-risk individuals earlier, potentially prompting lifestyle or pharmacological interventions to prevent or manage gout more effectively.

While this study demonstrates strong predictive performance, several limitations should be considered. First, the reliance on data from a specific population, in this case, Taiwanese biobank participants, may impact the generalizability of our findings. Second, the diagnosis of gout was based on self-report and uric acid levels: we did not utilize crystal confirmation for diagnosis. Additionally, although genetic data provided incremental benefits, further exploration of additional genetic markers beyond *ABCG2* rs2231142 might enhance prediction capabilities. Finally, the study’s reliance on propensity score matching, though helpful in controlling confounding, may not fully account for other unmeasured variables that influence gout risk. Future studies could explore machine learning models that incorporate additional environmental or lifestyle factors to capture a more comprehensive risk profile. Despite these, and given the escalating prevalence of gout, it is imperative to embrace innovative approaches, such as machine learning models. Integrating multidisciplinary input and establishing medical record networks,^7^ supported by advanced machine learning algorithms, can significantly enhance the precision and efficiency of gout management. Future studies incorporating diverse cohorts would enhance the external validity of our predictive model.

## Conclusions

In conclusion, the application of Random Forest and Gradient Boosting models demonstrates high accuracy in gout prediction, with or without the addition of genetic data. This study underscores the potential of machine learning in clinical prediction, particularly for conditions with complex metabolic and genetic underpinnings like gout. These findings pave the way for personalized risk assessment and preventive interventions, thereby advancing our ability to mitigate the burden of gout on public health.

## Data Availability

The data that support the findings of this study are available from Taiwan Biobank but restrictions apply to the availability of these data, which were used under license for the current study, and so are not publicly available. Data are, however available from the corresponding author, Prof. Yung-Po Liaw upon reasonable request and with permission of Taiwan Biobank.

## Abbreviation

RF, Random Forest; LR, Logistic Regression; AUC, area under the curve; DCA, decision curve analysis; PR, precision-recall; HDL, high-density lipoprotein; LDL, low-density lipoprotein; TG, triglycerides; APC, age-period-cohort; BMI, body mass index.
